# Assessment of Physical Activity Engagement and Promotion Among Cancer Survivors in Cardio-Oncology Care: Qualitative Study

**DOI:** 10.2196/104332

**Published:** 2026-09-11

**Authors:** Macy K Tetrick, Daniel Addison, Rujul Singh, Sara M St. George, Vanina Pavia, Anvitha Gogineni, Peter Washington, Sanam M Ghazi, Olivia Preston, James L Fisher, Roberto M Benzo

**Affiliations:** 1 Division of Cancer Prevention and Control, Department of Internal Medicine, College of Medicine, The Ohio State University, 3650 Olentangy River Rd, Columbus, OH, 43214, United States, 1-614-366-4652; 2 Cardio-Oncology Program, Division of Cardiology, The Ohio State University Medical Center, Columbus, OH, United States; 3 Cardio-Oncology Program, Division of Cardiology, University of Texas Southwestern Medical Center, Dallas, TX, United States; 4 Division of Epidemiology, College of Public Health, Ohio State University, Columbus, OH, United States; 5 Grossman School of Medicine, New York University, New York, NY, United States; 6 Sylvester Comprehensive Cancer Center, University of Miami Miller School of Medicine, Miami, FL, United States; 7 Department of Public Health Sciences, University of Miami Miller School of Medicine, Miami, FL, United States; 8 College of Medicine, The Ohio State University, , Columbus, OH, United States; 9 Division of Clinical Informatics and Digital Transformation (DoC-IT), Department of Medicine, University of California, San Francisco, San Francisco, CA, United States; 10 Arthur G. James Cancer Hospital and Richard J. Solove Research Institute, Columbus, OH, United States; 11 The Ohio State University Comprehensive Cancer Center, The Ohio State University Wexner Medical Center, Columbus, OH, United States

**Keywords:** cardio-oncology, physical activity, cancer survivorship, COM-B model, cardiovascular health, qualitative research, health behavior, exercise promotion, survivorship care, implementation science

## Abstract

**Background:**

Cardiovascular disease and cancer are leading causes of morbidity and mortality worldwide, with cancer survivors facing elevated cardiovascular risk due to shared risk factors and cardiotoxic treatments. Physical activity (PA) is a well-established strategy to improve cardiovascular health and survivorship outcomes; however, most cancer survivors do not meet recommended PA guidelines. Understanding multilevel influences on PA engagement and promotion is essential to improving implementation in cardio-oncology care.

**Objective:**

This study aimed to examine barriers and facilitators to PA engagement and promotion in cancer survivorship from both survivor and medical team perspectives using the Capability, Opportunity, Motivation-Behavior (COM-B) framework.

**Methods:**

A descriptive qualitative study was conducted with 12 cancer survivors and 12 medical team members involved in oncology care. Semistructured interviews explored experiences, perceptions, and practices related to PA across the cancer care continuum. Rapid qualitative analysis was used to identify themes, which were mapped to COM-B domains. Participants also completed surveys assessing PA, cardiovascular health (Life’s Essential 8 [LE8]), and sociodemographic factors.

**Results:**

Survivors had a mean age of 65.3 (SD 12.6) years and were evenly distributed by sex. PA engagement varied, with 42% (5/12) of survivors meeting recommended guidelines. The mean modified LE8-derived cardiovascular health score was 71.3 (SD 11.9), reflecting a standardized 6-domain composite (diet, PA, nicotine exposure, sleep, BMI, and blood pressure) calculated identically for all 12 participants. Across COM-B domains, physical limitation was the most commonly reported capability barrier (12/12 survivors), while current engagement in PA was the most prevalent capability facilitator (12/12 survivors). Opportunity was shaped by access to PA resources and social support, with social environment being the most frequently cited facilitator (12/12 survivors; 9/12 medical team members) and lack of access to PA resources being the most common barrier among medical team members (9/12). Motivation reflected perceived health outcomes as the most prevalent facilitator across both groups (12/12 survivors; 7/12 medical team members), alongside competing concerns such as safety and emotional responses. Medical team members’ ability to promote PA was influenced by survivor readiness, clinical context, and resource availability.

**Conclusions:**

PA engagement in cardio-oncology is shaped by dynamic interactions among capability, opportunity, and motivation across survivor and medical team member contexts. Although PA is widely valued, sustained engagement remains limited due to fluctuating symptoms, environmental constraints, and clinical uncertainties. Interventions should prioritize flexible, survivor-centered approaches and incorporate supportive clinical communication and resource alignment to enhance PA across the survivorship trajectory.

## Introduction

### Background

Cardiovascular disease (CVD) and cancer are the 2 leading causes of death worldwide, highlighting the need for strategies to improve health among individuals affected by both conditions [1]. Compared with individuals without cancer, people with a cancer diagnosis experience a significantly higher risk of CVD, attributable to shared cardiometabolic and behavioral risk factors, cancer-related physiological changes, and exposure to cardiotoxic therapies [2,3]. As cancer survival has improved over recent decades, CVD has emerged as a leading cause of morbidity and mortality among cancer survivors, with noncancer deaths, particularly cardiovascular-related deaths, surpassing cancer-related deaths between 1973 and 2012 [4,5].

Cardiotoxic cancer therapies (eg, anthracyclines, chest radiation, and immune-based agents) significantly contribute to both short- and long-term cardiovascular morbidity and mortality [6]. As a result, CVD has become a major determinant of survivorship outcomes, with cancer survivors who develop CVD experiencing significantly worse survival and greater morbidity over time [7]. Consequently, the 8-year overall survival rate was 60% among those who developed CVD compared with 81% among those who did not, highlighting the critical role of cardiovascular health in long-term cancer survivorship [7].

Given the central role of cardiovascular health in long-term cancer survivorship, identifying effective and scalable strategies to mitigate cardiovascular risk is a clinical priority. Physical activity (PA) represents one such strategy, with well-established benefits for cardiovascular health, functional capacity, and quality of life among individuals with cancer [8]. In a meta-analysis of controlled PA interventions in cancer survivors, PA was associated with significant improvements in cardiorespiratory fitness both during and after cancer treatment, with weighted mean effect sizes (Cohen *d*) of 0.51 and 0.65, respectively (*P*<.01) [9]. Regular engagement in PA among cancer survivors improves cardiorespiratory fitness and reduces cardiovascular risk, helping to counteract treatment-related deconditioning and loss of aerobic capacity. This is clinically important given that cardiorespiratory fitness is a strong and independent predictor of cardiovascular and all-cause mortality [8,10]. In addition to cardiometabolic benefits, the American College of Sports Medicine has published PA recommendations that have been linked to improvement in common cancer-related symptoms, including fatigue, anxiety, depression, poor health-related quality of life, lymphedema, and poor physical function [11].

Clinical guidelines from the American Cancer Society (ACS) and the American Institute for Cancer Research (AICR) recommend regular PA before, during, and after cancer treatment, with adults advised to engage in a minimum of 150 minutes of moderate-to-vigorous physical activity (MVPA) per week, alongside muscle-strengthening activities [8,12]. Despite these recommendations, population-based data demonstrate that the majority of survivors do not meet recommended PA guidelines [13]. Despite ample evidence supporting the benefits of PA, engagement and promotion remain inconsistent, shaped by factors operating at the survivor, medical team member, and health care system levels [14,15]. Together, these findings suggest that the role of PA in cancer care is shaped by a range of factors that can act as both barriers to and facilitators of participation and promotion.

Compared with general oncology populations, patients receiving cardio-oncology care face actual or potential cardiovascular complications of cancer treatment in addition to cancer-related morbidity. PA may therefore be relevant to both cancer survivorship and cardiovascular health. Survivors may also have difficulty determining whether symptoms reflect cardiotoxicity, cancer treatment effects, or normal exertion, creating uncertainty about safe PA participation [16]. Further, promoting PA in cardio-oncology may require coordination between oncology and cardiology teams for counseling, safety guidance, and referral to exercise services [17]. These features make cardio-oncology a clinically distinct setting for examining the multilevel influences on PA engagement and promotion.

These multilevel influences highlight the need for a behavioral framework capable of integrating survivor, medical team member, and health care system determinants of PA engagement in cancer care. The Capability, Opportunity, Motivation-Behavior (COM-B) model provides a comprehensive framework for understanding and influencing health-related behaviors by explicitly identifying modifiable conditions required for a behavior to occur. Within this model, behavior is conceptualized as the result of interactions among individuals’ physical and psychological capabilities, the social and environmental opportunities available to them, and both reflective and automatic motivational processes [18]. Applied to PA in cancer care, the COM-B framework offers a structured way to examine how survivor-level factors (eg, symptoms and beliefs), medical team member factors (eg, knowledge and time), and health care system factors (eg, access and resources) shape whether PA is initiated, sustained, and promoted.

Few studies have examined the influences of both survivor and medical team member factors in an integrated, theory-informed manner or explored how they are experienced and navigated within clinical interactions. Limited qualitative work has simultaneously incorporated survivor and medical team perspectives to understand how PA engagement and promotion unfold across survivorship. As a result, there remains an incomplete understanding of the specific barriers, facilitators, and contextual conditions that shape PA as both a survivor behavior and a clinical practice.

Prior research suggests that PA engagement in cancer survivorship is shaped by not only individual factors but also clinical and system-level influences. Although clinicians widely endorse PA, gaps remain in how it is assessed, discussed, and supported in practice, with uncertainty regarding roles, time constraints, and available referral pathways limiting consistent promotion. Survivors also report interest in receiving guidance from their care teams, yet counseling and support vary across settings [19]. Existing qualitative work has largely focused on survivor experiences alone, with less attention to how survivor and medical team member perspectives intersect within clinical care. As a result, there remains limited understanding of how PA is both experienced by survivors and operationalized in clinical interactions.

### Objectives

To address this gap, this qualitative study uses the COM-B framework to examine PA engagement and promotion in cancer survivorship from both survivor and medical team perspectives. By exploring how capability, opportunity, and motivation interact across roles and contexts, this study aims to generate actionable insights to inform more responsive clinical practice and intervention design for promoting and supporting PA throughout the survivorship trajectory.

## Methods

### Study Design

We conducted a descriptive qualitative study guided by the COM-B framework to examine PA engagement and promotion in the context of cancer treatment and survivorship. Semistructured interviews were used to explore perceived barriers and facilitators influencing PA behaviors among cancer survivors and medical team members involved in cancer care. The COM-B framework was used as an organizing lens to structure the examination of barriers and facilitators of PA behavior across medical team personnel (in terms of promotion) and cancer survivors (in terms of engagement).

In addition, cancer survivor participants completed a brief survey to capture self-reported sociodemographic characteristics, PA, and overall health measures. PA was assessed using the Rapid Assessment of Physical Activity (RAPA), a validated self-report measure of PA levels in adults [20]. The RAPA includes a series of yes/no items reflecting increasing levels of aerobic PA, with scores ranging from 1 (sedentary) to 7 (active). The final RAPA score was assigned based on the highest affirmed item. Consistent with the validated scoring criteria, participants with scores of 6 or higher were classified as “active.” Item 6 of the RAPA indicates engaging in at least 30 minutes of moderate PA on 5 or more days per week, whereas item 7 indicates engaging in at least 20 minutes of vigorous PA on 3 or more days per week. The RAPA also includes items assessing strength and flexibility activities, asking whether participants engage in muscle-strengthening activities (eg, lifting weights and calisthenics) and flexibility activities (eg, stretching and yoga) at least once per week. These items are scored separately from the RAPA aerobic score and were not incorporated into the aerobic score or the study’s classification of “active” status, which was based solely on the aerobic component. They are reported here descriptively. In addition to the RAPA, survivors reported weekly minutes of moderate or greater intensity activity as part of the PA domain of the Life’s Essential 8 (LE8) cardiovascular health assessment (described below). This continuous self-report item was classified against the ACS/AICR guideline of 150 or more minutes per week, providing a second, independently derived measure of guideline-consistent PA. Because the RAPA and this continuous minutes-based measure use different item formats and scoring approaches, the resulting classifications may differ for some participants.

Select social determinants of health, including income, education, and employment status, were assessed using the Protocol for Responding to and Assessing Patients’ Assets, Risks, and Experiences (PRAPARE) [21]. The full PRAPARE domain set was not analyzed for this manuscript.

Cardiovascular health was assessed using the American Heart Association’s LE8 framework, which results in a composite score based on 8 health behaviors and factors: diet, PA, nicotine exposure, sleep health, BMI, blood lipids, blood glucose, and blood pressure [22]. The diet component was operationalized using a Mediterranean diet–based scoring approach, consistent with the Modified Mediterranean Eating Pattern for Americans (MEPA) recommended within the LE8 framework [22,23]. When individual LE8 components were missing, composite scores were calculated using the 6 domains available for all participants (diet, PA, nicotine exposure, sleep, blood pressure, and BMI). Blood glucose was not included in the survey instrument and therefore was not assessed. Blood lipid data were available for only 4 of 12 participants (33%) due to missing responses. Both domains were therefore excluded from the primary composite and are reported descriptively where available. Given the small sample size, validated imputation approaches were not considered appropriate to address missing domain data. Because some participants were unable to report clinical measures, survey-derived health measures were analyzed descriptively to characterize overall health status within the sample.

All 12 survivor participants completed the full survey battery. Blood lipid values were collected via self-report. Some participants left this item blank or did not respond, resulting in missing data for this domain. Blood glucose was not included in the survey instrument and was therefore not assessed for any participant.

The target behavior for this analysis was engagement in and promotion of PA throughout cancer survivorship. Because PA behaviors are shaped by complementary roles within the clinical relationship (survivors engaging in PA and medical team members providing encouragement and guidance), both survivor and medical team perspectives were included.

### Participants and Recruitment

Survivor participants (n=12) were adults aged 18 years or older who had received a cancer diagnosis and were currently undergoing or had previously undergone cancer treatment involving cardiotoxic therapies, including targeted, biologic, radiation, or immune-based treatments. Medical team members (n=12) were health care professionals involved in the care of oncological survivors. Eligible survivor participants were required to have access to a device capable of supporting remote interviews (eg, smartphone, computer, or tablet) and to self-report interest in tracking cardiovascular symptoms and engaging in PA. Individuals who were pregnant, incarcerated, or unable to complete the interview in English were excluded.

Survivors were recruited through clinician referrals within a cardio-oncology clinic. Clinicians introduced the study to eligible survivors during routine appointments, and interested individuals were contacted by the research team to provide additional information and confirm eligibility. Medical team participants were recruited via professional referrals and word of mouth. All participants provided informed consent prior to enrollment.

### Data Collection

Semistructured interviews were conducted with research staff members remotely via videoconferencing platforms (eg, Zoom and Microsoft Teams) between December 2023 and July 2024. Interviews were conducted by the lead author (MKT) and/or the study’s principal investigator (RMB). Both were trained in rapid qualitative analysis (RQA) and qualitative interviewing techniques. Neither interviewer had a prior relationship with survivor or medical team participants. All participants were referred to the research team by the cardio-oncology clinic rather than recruited directly by the interviewers. Reflexivity was supported through regular team meetings in which interpretations were discussed and reconciled collaboratively rather than by a single analyst. While each interview followed the semistructured interview guide, interviewers remained flexible and allowed participants to elaborate on relevant topics as they arose to ensure complete data capture.

The semistructured interview guides used to conduct both survivor and medical team member interviews are available in Multimedia Appendix 1. Survivor interview questions explored experiences engaging in PA before, during, and after cancer treatment; reasons for being physically active or not being physically active; and external factors that may help or hinder PA engagement. Medical team participants were asked about their roles, confidence, and approaches to encouraging PA among survivors. Prompts were aligned with COM-B domains, including capability (eg, symptoms and knowledge), opportunity (eg, time, access, and social support), and motivation (eg, beliefs and priorities). Survivor participants received a US $25 gift card upon interview completion.

Interviews lasted approximately 60‐90 minutes and were audio-recorded with participant consent. Most interviews were also video-recorded, although participants were given the option to keep their cameras off. Interviews were transcribed verbatim using a Zoom- or Teams-generated transcript and edited by a research team member when necessary. Survey data were analyzed descriptively to summarize participant characteristics, PA levels, cardiovascular health, and self-rated general health status.

### Data Analysis

Analysis followed the RQA approach by Hamilton and Finley [24], with attention to best practices for rigor and validity [25,26]. RQA, used to analyze interview data, is a team-based approach that emphasizes systematic review and synthesis of qualitative data to identify actionable themes [24,25]. This study used a hybrid, deductive-dominant analytic approach: the COM-B framework was applied a priori to structure both the interview guides and the RQA summarization process. Research team members were trained in the RQA process. One researcher completed an initial review of each transcript, summarizing key points directly into structured templates organized by the COM-B domain and subdomain. A second researcher subsequently reviewed the same transcript, with access to the initial summary, to compare interpretations. Because reviewers were not blinded to one another’s summaries, a formal interrater agreement statistic was not calculated. Discrepancies were resolved through team discussion and consensus. Within this deductively structured framework, the specific content of facilitator and barrier themes was generated inductively from participant data. Summaries were then organized into analytic matrices to enable comparison across participants and facilitate theme development. A template of the analytic matrix structure used for theme development is provided in Multimedia Appendix 2. Data collection and analysis occurred concurrently over approximately 1 year, with transcripts reviewed and summarized in a rolling manner as interviews were completed. As data collection and analysis progressed, the team observed that later interviews increasingly reinforced previously identified themes rather than introducing substantially new ones, consistent with thematic redundancy. Formal saturation metrics were not applied as a stopping criterion. Sample size was determined a priori based on the scope of the study. Final themes were mapped to the COM-B framework and categorized as barriers or facilitators within the overarching domains of capability, opportunity, and motivation. Consistent with the COM-B model, these domains were further specified as physical and psychological capability, physical and social opportunity, and reflective and automatic motivation. Representative quotations were selected to illustrate each theme. Sociodemographic characteristics of survivor participants were collected via a survey and summarized descriptively.

### Ethical Considerations

This study was approved by The Ohio State University Institutional Review Board (IRB #2021C0018). All participants provided informed consent prior to participation. Interview recordings and transcripts were deidentified, assigned a unique study ID, and stored securely in accordance with institutional policies.

## Results

### Participant Characteristics

Participant demographic and clinical characteristics derived from both survey and interview data are presented in Table 1. A total of 12 cancer survivors participated in interviews and completed surveys. Survivor participants had a mean age of 65.3 (SD 12.6) years, with an equal representation of women and men. Most survivors identified as White and non-Hispanic (10/12, 83%). BMI values indicated that the majority of survivors were either overweight (5/12, 42%) or obese (4/12, 33%), with a mean BMI of 26.9 (SD 4.53) kg/m². Educational attainment was generally high, with 58% (7/12) of participants reporting completion of a graduate or professional degree. Most survivors (10/12, 83%) were retired or receiving disability benefits, and annual household income most frequently fell between US $100,000 and $199,999 (5/12, 42%). Cancer diagnoses were heterogeneous. The most commonly represented diagnosis was acute myeloid leukemia (3/12, 25%), followed by breast cancer (2/12, 17%), with several other malignancies represented in smaller numbers. Most survivors reported exposure to multiple cancer-directed therapies, reflecting complex treatment histories. These included chemotherapy (6/12, 50%), radiation therapy (5/12, 42%), tyrosine kinase inhibitors (4/12, 33%), stem cell transplantation (4/12, 33%), hormonal therapy (3/12, 25%), and surgery (3/12, 25%), with fewer participants reporting exposure to immune checkpoint inhibitors (1/12, 8%) or other treatment modalities (4/12, 33%).

**Table 1.** Demographic and clinical characteristics of the sample population.

Variable		Survivors (n=12)	Medical team members (n=12)
Age (years), mean (SD)		65.3 (12.6)	37.8 (6.4)
Time in position (years), mean		—^a^	4.5
Sex, n (%)			
Female		6 (50)	7 (58)
Male		6 (50)	5 (42)
Race or ethnicity, n (%)			
White, non-Hispanic		10 (83)	6 (50)
White, Hispanic		0 (0)	1 (8)
Black or African American		1 (8)	1 (8)
Asian		0 (0)	3 (25)
Multiple races or ethnicities		1 (8)	1 (8)
BMI (kg/m^2^), n (%)			
18.5‐24.9		3 (25)	—
25‐29.9		5 (42)	—
≥30		4 (33)	—
BMI, mean (SD)		26.9 (4.53)	—
Employment^b^ (most common category), n (%)			
Retired or disabled		10 (83)	—
Education^b^ (most common category), n (%)			
Graduate or professional degree		7 (58)	—
Annual income^b^ (most common category), n (%)			
US $100,000-199,999		5 (42)	—
Job position, n (%)			
Cardio-oncologist		—	4 (33)
Oncologist		—	3 (25)
Radiation oncologist		—	1 (8)
Nurse practitioner		—	1 (8)
Physical therapist		—	1 (8)
Exercise physiologist		—	1 (8)
Dietitian		—	1 (8)
Cancer type, n (%)			
Acute myeloid leukemia		3 (25)	—
Breast		2 (17)	—
Prostate		1 (8)	—
Lymphoma		1 (8)	—
Multiple myeloma		1 (8)	—
Uterine		1 (8)	—
Chronic lymphocytic leukemia		1 (8)	—
Lung		1 (8)	—
Neuroendocrine malignancy		1 (8)	—
Cancer treatment received, n (%)			
Chemotherapy		6 (50)	—
Radiation		5 (42)	—
Stem cell transplantation		4 (33)	—
Tyrosine kinase inhibitors		4 (33)	—
Hormonal therapy		3 (25)	—
Surgery		3 (25)	—
Immune checkpoint inhibitors		1 (8)	—
Others		4 (33)	—

a Not collected or not applicable.

b Complete education, employment, and annual income data are provided in Table S2 in Multimedia Appendix 3.

In addition, 12 medical team members involved in the care of survivors with cancer completed interviews. Medical team members had a mean age of 37.8 (SD 6.4) years and reported an average of 4.5 years in their current professional roles. Of the 12 medical team members, 7 (58%) identified as female, and the team represented a range of racial and ethnic backgrounds, including White and non-Hispanic (6/12, 50%), Asian (3/12, 25%), White and Hispanic (1/12, 8%), Black or African American (1/12, 8%), and multiple racial or ethnic identities (1/12, 8%). Professional disciplines included cardio-oncology physician (4/12, 33%), medical oncologist (3/12, 25%), and radiation oncologist (1/12, 8%), as well as allied health professionals such as nurse practitioner (1/12, 8%), physical therapist (1/12, 8%), exercise physiologist (1/12, 8%), and dietitian (1/12, 8%).

### Self-Reported PA

Based on self-reported weekly PA minutes collected as part of the LE8 PA domain, 42% (5/12) of survivor participants met recommended PA guidelines (≥150 min per week of MVPA), while 58% (7/12) did not. Using the separate, categorical RAPA instrument, 50% (6/12) of participants were classified as active, while 50% (6/12) were classified as underactive. Of the 12 participants, 9 (75%) were classified concordantly across the 2 measures: 4 met both criteria and 5 met neither; the remaining 3 were classified differently. These 2 measures rely on different item structures and scoring algorithms and are not expected to yield identical classifications for all participants.

For descriptive purposes only, and independent of the RAPA aerobic score reported above, 67% (8/12) of survivor participants reported engaging in strength training activities at least once per week, and 50% (6/12) reported engaging in flexibility activities at least once per week. Categorized jointly, 42% (5/12) reported both, 33% (4/12) reported one only, and 25% (3/12) reported neither.

The mean cardiovascular health score (modified LE8-derived score) was 71.3 (SD 11.9). Descriptive details, including LE8 domain-level scores, are provided in Table S1 in Multimedia Appendix 3.

### COM-B Themes

The results are organized according to the COM-B framework, capturing themes related to capability, opportunity, and motivation across both survivor and medical team member perspectives. Themes and subthemes identified within each domain are summarized in Table 2. In the text below, we describe these themes in greater detail, using representative quotations to illustrate how participants experienced and interpreted PA engagement and promotion in cancer survivorship. A table of additional quotes representing each theme can be found in Multimedia Appendix 4.

**Table 2.** Summary of the domain system (interview data).

COM-B^a^ domain, facilitator/barrier, and subdomain	Survivor interview theme (n)	Medical team member interview theme (n)
Capability		
Facilitator		
Physical	Current engagement in PA^b^ (n=12)	Survivor self-efficacy (n=3)
Psychological	Knowledge and awareness of PA levels and ability (n=9)	Knowledge and awareness of survivor PA levels and ability (n=9)
Barrier		
Physical	Physical limitations (n=12)	Survivors’ symptom- or treatment-related burden (n=2)
Psychological	—^c^	Lack of knowledge on survivor PA levels (n=3)
Opportunity		
Facilitator		
Physical	PA resources (n=11)	Online PA resources (n=4)
Social	Supportive social environments (classes, family, and friends) (n=12)	Supportive social environments for survivors (n=9)
Barrier		
Physical	Weather and time (n=7)	Lack of access to PA resources (n=9)
Motivation		
Facilitator		
Reflective	Perceived health outcomes (n=12) PA-related resources (n=7) Intentions to be active (n=7)	Perceived health outcomes (n=7) Survivors’ inclination to be active (n=3)
Automatic	Emotions surrounding PA (n=5) Routine or habit (n=7)	—
Barrier		
Reflective	Physical limitations (n=8) Safety concerns, resource preferences (n=3)	Treatment-related limitations (n=4)
Automatic	Emotions surrounding PA (n=7)	Survivors’ emotions surrounding PA (n=3)

a COM-B: Capability, Opportunity, Motivation-Behavior.

b PA: physical activity.

c Not applicable or not available.

### Domain: Capability

#### Overview

In the COM-B framework, capability refers to an individual’s capacity to engage in a behavior and encompasses (1) physical capability (eg, physical strength, stamina, and symptom burden) and (2) psychological capability (eg, knowledge, understanding, and cognitive processes required to perform the behavior) [18]. In this study, capability captured factors influencing whether survivors were physically able and cognitively prepared to engage in PA, as well as whether medical team members felt equipped to assess and support PA participation.

#### Physical Capability

We identified 1 theme under physical capability: the *ability to engage in PA*. Every survivor mentioned their current engagement in activities and their ability to perform activities of daily living, such as household chores, which demonstrated their physical capability to stay active. One survivor mentioned:

*I’m more of a person that takes on the chores of life. And that’s where I get a lot of my physical activity in.*

For some, this baseline level of PA served as a foundation for further engagement in PA. At the same time, all survivor participants noted physical limitations, such as symptom burden and decreased fitness levels, that presented significant barriers to their ability to regularly engage in PA. One survivor noted:

*There’s been incidences during the whole time where I couldn’t be very active… I've seen a lot of muscle atrophy set in. I’ve got very weak and things like that.*

If survivors were capable and self-driven to engage in PA, it facilitated medical team members’ ability to promote PA. However, symptom-driven limitations also shaped medical team members’ approaches, as they could only prescribe PA that aligned with what survivors’ physical conditions allowed. One medical team member explained:

*Every cancer patient is so different, and they have their own limitations and physical limitations.*

#### Psychological Capability

We identified 1 theme under psychological capability: the role that *knowledge and awareness* play in PA from survivors’ and medical team members’ perspectives. Many survivors mentioned their previous experience with PA, facilitating them with the cognitive tools to plan and engage in PA. Similarly, most medical team members emphasized that their knowledge of the benefits of PA and tips and tricks to help promote PA were key facilitators. On the other hand, some medical team members noted that a lack of knowledge regarding individual survivors’ current PA levels and activity history hindered their ability to tailor and promote PA effectively. One medical team member stated:

*People are super inexperienced on exercise, so the more information they have, the more successful they'll be with sticking with it.*

### Domain: Opportunity

#### Overview

Within the COM-B framework, opportunity refers to external factors that enable or prompt behavior and includes (1) physical opportunity, such as environmental resources, access, time, and contextual constraints, and (2) social opportunity, including interpersonal influences, social norms, and support systems [18]. In this study, opportunity captured how features of survivors’ physical environments and social contexts shaped their ability to engage in PA, as well as how these same conditions influenced medical team members’ ability to promote PA.

#### Physical Opportunity

The first theme identified was the importance of *PA resources* for both medical team members and survivors regarding PA. Most survivors made note of access to public environments (eg, parks and gyms) along with tools, such as home PA equipment, online resources (eg, apps and YouTube), workout plans, and fitness trackers, as facilitators to engage in PA. Despite the availability of PA resources described by some participants, self-reported findings indicated that a majority of participants (7/12, 58%) did not meet recommended PA levels. Medical team members also identified access to online tools as a key facilitator for promoting PA among cancer survivors, noting that these resources expand accessible and flexible options for engaging in PA. One survivor mentioned:

*I do think that like having things that you can do from the comfort of your own home is very important, because sometimes you don't feel like…looking like a normal person…on the flip side…gym accessibility would be awesome.*

However, several medical team participants also discussed that a *lack of access* to these resources is a barrier to promoting PA among survivors. Medical team members recognized that a lack of access to public parks or gyms, or tools and digital resources, reduces the opportunities for survivors to engage in regular PA. One medical team member noted:

*…you want appropriate people doing the videos, not just someone,…that’s a star on YouTube…Someone that’s qualified is really important because our survivors are…receiving chemotherapy. They may not be in the best shape, and it’s important to have the right…movements and things like that.*

Another medical team member stated:

*You can only do so much. I mean, I can't get them a pair of running shoes. I can't get them a membership at the gym, or make it warmer outside to run, or whatever it is that would actually work for them.*

Another theme identified was *weather and time* regularly engaging in PA. Lack of time and poor weather conditions can be limiting factors in engaging in PA. One survivor stated:

*I mean, it’s harder in the winter time to walk and stuff like that…That’s the hard time. It’s hard to walk.*

#### Social Opportunity

Social support emerged as an integral facilitator for PA from both survivor and medical team member perspectives. For patients, access to local *classes, events, and support groups* created a sense of community and accountability, ultimately making engaging in PA more enjoyable. Many survivors also emphasized the supportive role that *their family, friends, pets, and medical team* provide in engaging in regular PA. Medical team members similarly highlighted the importance of supportive social environments to facilitate promoting PA among survivors. One medical team member mentioned:

*Their families are the biggest cheerleaders…I have a lot of survivors…who are part of exercise groups or walking groups…and their goal is to get back to being with those people.*

### Domain: Motivation

#### Overview

Within the COM-B framework, motivation refers to the internal processes that direct and sustain behavior and includes (1) reflective motivation, such as conscious intentions, beliefs about outcomes, and evaluations of risk, and (2) automatic motivation, including emotional responses, habits, and routines [18]. In this study, motivation captured how survivors’ beliefs, intentions, emotions, and habitual patterns influenced their engagement in PA, as well as how medical team members’ perceptions of survivor motivation shaped their approaches to promoting PA.

#### Reflective Motivation

The first theme identified was the relationship between PA and *perceived health*. Both survivors and medical team members noted that the benefits of PA (eg, symptom relief and appearance) served as a motivating factor for engaging in and promoting PA. However, both groups also identified barriers to PA. Survivors reported that physical limitations, such as fatigue and discomfort, reduced their motivation to engage in regular PA. Medical team members echoed these concerns, noting that survivor-specific limitations related to treatment and disease often constrained their motivation to promote PA. One survivor noted:

*Today, coughing and breathing is tough…I was thinking about getting on the bike…maybe trying for… 10 minutes or so. But usually, I'd like to go 30 minutes on the bike, if I can, but we'll see…if I get things moving and agitated then I start coughing a lot.*

Another survivor explained:

*I feel like even in terms of physical appearance, again, there’s so many things that change, but being able to still feel good about my body and like how I present myself is important to me.*

One medical team member stated:

*It’s extremely important. It’s directly linked to their cardiovascular outcomes and their oncology outcomes…the physical activity is critical. I try to encourage all my survivors to be as physical as they can be.*

A second theme identified was the factors associated with PA-related resources. Several survivor participants reported that having access to at-home workouts and digital health tools was motivating. Many participants appreciated the flexibility that home workout options provided. This approach reduced logistical barriers, allowing them to engage in PA. At the same time, PA tracking tools provided motivating metrics, such as step count, that allowed survivors to understand and manage their PA. One survivor highlighted:

*I have a lot of apps on my iPad about exercise and different things, so those kind of help a little bit. I will open up one and say, “Okay, let me see if I can get a 10-minute workout in or a 5-minute workout in.”*

However, the positive influence of PA-related resources was tempered by *resource preferences and safety concerns*, which limited motivation to engage in PA. Few survivors were reluctant to use digital resources, such as online PA programs or tracking apps, due to discomfort with technology or a preference for more traditional methods. Moreover, some survivors expressed caution about the risks associated with PA, such as potential injury or the possibility of exacerbating symptoms, increasing their apprehension about engaging in PA. This focus on safety reflects survivors’ conscious assessments of their physical limitations and the potential health consequences of PA. One survivor noted:

*…during treatment, you’re really afraid that you might hurt something or break something, that you're fragile and frail. It’s pretty easy to say, “I'll wait till after treatment…”*

Several survivor participants also highlighted their *intentions* to engage in PA, which included a positive outlook toward PA and creating strategies to maintain accountability. Survivors noted that their motivation to engage in PA was influenced by the setting of goals and plans. Medical team members also observed this inclination in some survivors, stating that a survivor’s motivation (eg, intentions, perceived beliefs, and consequences) to engage in PA serves as a key facilitator in promoting PA in survivors. One survivor explained:

*Before that, I could bench press the 25 pounds. It’s like, I wanna get back to that. I can do 20 now, but I can't do 25 yet.*

#### Automatic Motivation

The first theme identified was the positive and negative influence that a survivor’s *emotions* have on their PA behaviors. On one hand, some survivors described experiencing positive feelings associated with engaging in PA, which was a facilitating factor. On the other hand, some survivors described negative feelings associated with engaging in PA, thus acting as a barrier. Aligned with this finding, medical team members also noted that the association of survivors’ emotional relationship with PA influenced their motivation to promote PA. One medical team member noted:

*…the biggest thing is their own motivation, like if they’re not motivated to exercise or to do any physical activity, they’re not gonna do it.*

One survivor mentioned:

*When I’m achy and stiff, I’ll go to a stretching type class, and I’ll just feel better. I also feel when I'm kind of groggy, I’ll go to some of these classes and just getting my blood flowing…my mood improves. I just feel better.*

The second theme identified was the *importance of a routine or habit* as a positive influence on motivation. Survivors noted that once PA became part of their daily routine, it required less conscious decision-making, which reinforced their ability to maintain regular PA. One survivor stated:

*Just doing it as part of a routine. Say, this is my routine. I get up. I find the class that I want to go to at the center. I click on it and I go to it.*

## Discussion

### Principal Findings

In this qualitative study informed by the COM-B framework, findings indicate that although PA is widely valued, consistent engagement remains limited. Although participants frequently described access to PA resources, less than half (5/12, 42%) met the recommended PA guidelines, suggesting a disconnect between available resources and sustained engagement. Rather than reflecting a lack of interest or intent, this pattern highlights the conditions under which PA requires adaptation, reassurance, and contextual support.

Capability-related factors included fluctuating symptoms, knowledge and awareness of PA, and readiness for PA, which shaped both survivors’ ability to engage and medical team members’ approaches to promoting PA. Opportunity-related influences reflected access to resources and supportive environments, where availability of PA options and social support facilitated engagement, while practical constraints, such as time and environmental barriers, limited feasibility. Motivation operated through both reflective processes, including weighing perceived health benefits alongside safety concerns and intentions to be active, and automatic processes, such as emotional responses and established routines.

The COM-B model conceptualizes behavior as arising from an interacting system of capability, opportunity, and motivation, with capability and opportunity also influencing motivation [18]. Participants’ accounts illustrated these cross-domain relationships. Medical team members described family support and peer exercise groups as strengthening survivors’ motivation by connecting PA with valued relationships and social roles, illustrating how social opportunity could support reflective motivation. Survivors described treatment-related physical vulnerability as contributing to perceived risk and fear-based avoidance, suggesting that limitations in physical capability could also weaken reflective motivation. These interactions varied across participants rather than reflecting a single, uniform pathway.

Descriptive survey findings complemented these themes by demonstrating variability in PA engagement, with fewer than half of participants (5/12, 42%) meeting recommended PA guidelines and only half (6/12, 50%) classified as active based on the RAPA. Many participants expressed motivation to engage in PA, suggesting that motivation alone may be insufficient to sustain engagement in some contexts, particularly in the absence of supportive conditions or resources. This aligns with prior evidence showing that adherence to PA guidelines among cancer survivors remains low, with previous studies reporting that only 20% meet aerobic PA guidelines [27]. Self-reported health status similarly varied, reinforcing heterogeneity in overall health. Together, these results underscore that PA engagement among survivors and promotion from medical team members represent complex, role-specific behaviors shaped by interacting influences.

### Comparison With Prior Literature

These patterns are consistent with prior research suggesting that PA engagement across cancer survivorship is shaped by interrelated conditions that influence feasibility and support. Prior survivorship studies have similarly emphasized that engagement in health-promoting behaviors following a cancer diagnosis is influenced by dynamic and context-dependent situations. For example, qualitative reviews of survivor perspectives describe PA engagement as contingent on a range of factors, including symptoms, environmental conditions, and the variability of reassurance and guidance from health care professionals [28]. With most cancer survivors not achieving recommended PA levels, this reflects ongoing challenges related to clinical practice, resource availability, and behavior change across both survivor and medical team member contexts [19].

Several specific themes identified in our study closely align with those reported in qualitative syntheses of cancer survivors’ experiences with PA. Among adults (n=12) with cancer seen in the cardio-oncology clinic, treatment-related side effects, particularly fatigue, emerged as major barriers to PA participation. This finding is consistent with prior research identifying treatment-related symptoms, including fatigue, as predominant barriers to engaging in PA [28,29]. Similarly, environmental concerns were identified as barriers, aligning with findings from qualitative syntheses and systematic reviews, including weather conditions, lack of suitable or adapted PA environments, and financial constraints [28,30]. Psychological concerns were prominent, with fears of worsening symptoms or disease and reduced self-esteem or motivation shaping survivors’ willingness to engage in PA, consistent with prior literature describing emotional and cognitive barriers to PA [30]. Support-related factors also emerged as key facilitators, as participants emphasized the value of encouragement, structure, guidance from health care professionals, and group-based settings in supporting PA engagement, alongside frustration when such support was absent, which has been similarly reported in qualitative studies and mixed-method reviews of cancer survivors [28,30].

Prior literature has documented that clinicians’ promotion of PA in cancer care is shaped by multiple professional and contextual considerations, rather than by endorsement of PA alone. Qualitative studies of oncology care medical team members indicate that, although clinicians widely recognize the benefits of PA, promotion is influenced by factors such as role clarity, confidence and training, time constraints, availability of referral resources, and concerns related to survivor symptoms and safety [31]. Complementing these findings, Schmitz et al [19] indicated that clinicians often lack clarity regarding their role in assessing, advising, and referring patients for PA, alongside system-level barriers such as limited referral pathways, insufficient resources, and challenges integrating PA promotion into routine care. Importantly, this work emphasizes that oncology clinicians are not expected to provide detailed PA prescriptions but instead play a key role in encouraging PA and directing survivors to appropriate professionals and programs (eg, cancer rehabilitation services, exercise physiologists, and community-based programs), highlighting the need for accessible referral pathways within cancer care [19].

Collectively, prior work shows that both survivor engagement and clinician promotion of PA are shaped by aligned, or misaligned, conditions within the clinical encounter. This is consistent with a recent qualitative study applying the COM-B framework and Theoretical Domains Framework to examine PA integration in breast cancer care from both patient and health care professional perspectives, which similarly found that patient-level barriers (eg, misconceptions and fatigue) and professional-level barriers (eg, need for clearer protocols and training) shape integration of PA into routine oncology care [32]. By integrating survivor and medical team member perspectives, this study shows that PA engagement is not simply an individual survivor behavior but a negotiated clinical behavior that is jointly experienced and navigated throughout the survivorship journey.

### Implications for Intervention Design

Findings from this study indicate that promoting PA in cancer survivorship may benefit from flexible, responsive approaches rather than relying on standardized or one-time recommendations, as both survivors and medical team members described PA engagement as fluctuating over time. These findings highlight the potential value of interventions that facilitate ongoing communication between survivors and medical team members, allowing recommendations to be tailored and revisited as clinical circumstances change. Such approaches may be particularly relevant in clinical contexts where PA has the potential to influence patient outcomes, including treatment tolerance, recovery, and rehabilitation.

For survivors who face symptom burden, safety concerns, or environmental barriers, future intervention development could examine adapted home-based programs that provide feasible alternatives to traditional PA settings. This approach is supported by evidence that home-based PA interventions can provide accessible alternatives for cancer survivors facing feasibility barriers and that interventions incorporating frequent counseling are associated with larger improvements in fatigue, suggesting the importance of pairing flexible PA resources with ongoing support that can be revisited and adapted [33]. These results indicate that interventions should support clinicians in translating general PA guidance into feasible, individualized recommendations that account for survivors’ potential barriers. Consistent with this approach, evidence from implementation research suggests that strategies incorporating individualized and interactive elements, such as tailored counseling and active survivor-medical team member communication, are more effective at increasing PA uptake during and after cancer treatment than one-time or generic recommendations [34]. However, the feasibility of delivering individualized counseling in routine oncology care may be limited by time constraints, staff availability, and variability in referral resources. To address this gap, future interventions should examine low-burden implementation strategies that preserve individualized support while fitting within existing clinical workflows.

Access to resources also emerged as an important factor shaping PA engagement. Survivors frequently described community spaces, home-based options, and digital tools as facilitating adaptation and continuity of PA, while medical team members noted variability in survivors’ access to such resources as a consideration during promotion. Both survivors and medical team members described the supportive role that survivors’ social environments, including family, friends, and their medical team, can play in facilitating physical engagement. This aligns with qualitative evidence demonstrating that social support functions as a key facilitator of PA among cancer survivors, enabling both adoption and maintenance of PA through mechanisms such as encouragement, accountability, and companionship [35]. In this context, clinicians may play a supportive role by helping survivors identify and make use of feasible, accessible resources, both material and social, that align with their circumstances. Framing PA promotion as an adaptive clinical process that incorporates individualized guidance and practical support may enable more sustained engagement without increasing burden within routine care. Future interventions should examine how resource-matching strategies, including digital PA tools, wearable-supported self-monitoring, group-based remote programs, and curated community referral options, can be tailored to survivors’ needs and integrated into survivorship care without increasing burden on survivors or medical team members.

The findings also point to important considerations for the design of PA interventions in cancer survivorship. Interventions that focus exclusively on modifying survivor behavior may be limited if they do not also account for the clinical interactions through which PA is discussed and supported. The results suggest that intervention approaches should allow for flexibility, recognizing that survivor capacity and circumstances often change over time rather than remaining stable. Given that participants described clinical encounters as key settings where PA is discussed and negotiated, future intervention development should examine approaches that account for the barriers identified in our study, including symptom burden, safety concerns, competing demands, environmental constraints, social support needs from family and medical team members, and variability in clinical guidance. Population-based evidence indicates that fewer than half of cancer survivors report receiving PA counseling from a medical team member, yet those who do receive counseling are more likely to engage in higher levels of PA, highlighting the survivor-medical team member interaction as a meaningful opportunity for intervention [36].

Intervention approaches may also benefit from attending to how counseling is translated into action, including connection to feasible PA resources and tools, such as home-based options, community spaces, and digital supports. Additionally, leveraging survivors’ existing social environments may further support engagement and align with evidence that social support facilitates adoption and maintenance of PA among cancer survivors [35]. Overall, interventions that align survivor experiences, clinician guidance, and available supports are more likely to be effective than those targeting any single element in isolation.

### Implications for Future Research

Future research should continue to examine PA engagement and promotion as dynamic, context-dependent processes that unfold throughout survivorship. Longitudinal qualitative or mixed-methods studies may be particularly valuable for understanding how survivors’ and clinicians’ assessments of feasibility, support needs, and priorities shift over time in response to changing symptoms, treatment phases, and life circumstances. Studies that intentionally integrate survivor and medical team perspectives within shared care contexts could further illuminate how clinical interactions shape PA engagement. Larger-scale qualitative or mixed-methods studies, including those conducted across multiple clinical settings or health systems, would be useful for assessing the transferability and generalizability of these findings to more diverse survivorship populations. In addition, research that evaluates strategies designed to support survivor-medical team member PA counseling within routine care may help strengthen translation of evidence into practice. Greater attention to variability across care settings, resource availability, and survivor populations will also be important for informing equitable and scalable approaches to PA promotion in survivorship.

### Implications for Digital Health and Referral Pathways

Given the relevance of digital health approaches to supporting PA in survivorship, several implications for digital tools and referral pathways emerged from this study. Both survivors and medical team members described interest in accessible referral pathways connecting survivors to appropriate professionals and programs (eg, cancer rehabilitation services, exercise physiologists, and community-based programs) [19]. Recent feasibility studies offering PA interventions provide examples of such approaches, including brief PA communication and clinician referral pathways involving exercise professionals, as well as electronic triage tools paired with brief counseling and referral to PA or rehabilitation services [37,38]. For survivors facing symptom burden, safety concerns, or environmental barriers, digital and technology-supported PA resources, including group-based videoconference (tele-exercise) programs, wearable technology–based interventions, and app-based tracking tools, offer feasible and scalable alternatives to traditional PA settings [39,40]. Future interventions should examine how resource-matching strategies, including digital PA tools, wearable-supported self-monitoring, group-based tele-exercise programs, and curated community referral options, can be tailored to survivors’ needs and integrated into survivorship care without increasing burden on survivors or medical team members.

### Strengths and Limitations

This study has several strengths. First, it incorporated both survivor and medical team perspectives from a single setting, providing a more comprehensive view of PA engagement and promotion in cancer survivorship than studies focused on a single group. Examining these perspectives together enabled the identification of shared challenges and complementary insights within clinical interactions. Second, the focused, theory-informed interview structure supported in-depth exploration of PA engagement and promotion within a cardio-oncology setting, where PA may be shaped by not only cancer survivorship concerns but also cardiovascular risk, treatment-related symptoms, and perceptions of safety. The use of the COM-B framework to guide data collection and analysis supported systematic examination of capability, opportunity, and motivation as interacting influences on PA engagement and promotion. Although the interview guide also addressed symptom tracking, those findings are reported separately in another manuscript. Additionally, descriptive survey data provided useful context regarding participants’ self-reported PA behaviors and cardiovascular health characteristics, although these data were not intended to validate or explain individual qualitative themes. Lastly, conducting interviews remotely helped reduce logistical and health-related barriers to participation, potentially increasing accessibility for individuals who may have had difficulty attending in-person interviews.

The interpretation of these findings should consider several limitations. First, the study relied on self-reported perceptions and experiences, which reflect participants’ accounts of PA engagement and promotion rather than directly observed behaviors or objectively measured outcomes. PA measures were self-reported and descriptive in nature, which may be subject to recall or reporting bias. The cardiovascular health composite reported here reflects a modified, 6-domain adaptation of the American Heart Association’s LE8 framework, excluding blood glucose (unavailable for all participants) and blood lipids (available for only 4 of 12 participants), and should not be interpreted as equivalent to the validated 8-domain LE8 score. A sensitivity analysis among the subset of participants with lipid data indicated that including this domain did not shift composite scores uniformly (mean change=1.0 points; range −3.1 to +4.4), suggesting that the original variable-denominator approach could have introduced variability tied to data availability rather than true cardiovascular health status. All interviews were conducted remotely, which may have shaped the nature of participant responses and introduced selection bias favoring individuals with greater comfort using technology or access to remote communication platforms. The modest gift card incentive (US $25) provided to survivor participants, while intended to offset time and participation burden, may have also influenced recruitment speed or introduced a degree of selection bias toward individuals more motivated by the incentive or with greater flexibility to participate. This potential influence was not formally assessed. The sample size was relatively small and, while appropriate for qualitative inquiry, may not capture the full range of perspectives across cancer types, treatment stages, or sociodemographic backgrounds. This diagnostic, treatment, and professional heterogeneity strengthened our ability to identify behavioral themes that generalize across clinical contexts, though it also precluded stratified analysis by diagnosis, treatment type, or professional role. Theme prevalence may vary across these subgroups. Survivor participants tended to have higher levels of income and educational attainment and were predominantly White and non-Hispanic, which may limit the transferability of the findings to more socioeconomically and racially or ethnically diverse survivorship populations. Barriers, such as weather, time, and gym access, reflect this relatively affluent sample. Structural social determinants of health barriers common in marginalized populations (eg, transportation, neighborhood safety, cost, and job flexibility) were unlikely to be captured and warrant a dedicated study. In addition, participants were recruited from a single clinical setting, which may limit transferability to other survivorship care environments with different resources, workflows, or practice norms. Relatedly, because participants were recruited from a specialized cardio-oncology clinic rather than a general oncology setting, both survivors and medical team members may have had greater baseline awareness of and engagement with lifestyle interventions than would be expected in general oncology populations, which may have influenced the prominence of themes related to knowledge, motivation, and support for PA. Finally, although the COM-B framework provided a useful structure for organizing data collection and analysis, its application may have highlighted certain behavioral influences while de-emphasizing others. Alternative approaches could have yielded different interpretive themes.

### Conclusion

This qualitative study explored how PA engagement and promotion are experienced and navigated in cancer survivorship from both survivor and medical team perspectives. Across interviews, PA was widely recognized as valuable, yet participation and promotion were influenced by changing symptoms, competing priorities, perceived safety, available resources, and the circumstances of clinical encounters. Patterns of PA were not static; instead, engagement reflected ongoing adjustment to health status and life context rather than simple adherence to recommendations. These findings highlight PA as a behavior that evolves over time within survivorship rather than one that can be adequately supported through singular or standardized guidance.

By applying the COM-B framework, this study offers insight into how capability, opportunity, and motivation interact across survivor and clinical contexts to shape PA engagement and promotion. The findings underscore the importance of clinical conversations, social and material supports, and adaptive approaches that accommodate fluctuation rather than expecting consistency. Attention to how PA is discussed, supported, and resourced may help bridge the gap between evidence and sustained engagement in daily life. Collectively, these insights contribute to a growing emphasis on flexible, context-aware strategies to support PA throughout the survivorship trajectory and may inform more responsive approaches to clinical practice, intervention design, and future research.

## Supplementary material

10.2196/104332 Multimedia Appendix 1 Medical team member and survivor participant interview guides.

10.2196/104332 Multimedia Appendix 2 Analytic matrix structure template.

10.2196/104332 Multimedia Appendix 3 Supplementary survey results.

10.2196/104332 Multimedia Appendix 4 Representative quotes from medical team members and survivor participants.

## Abbreviations

ACS: American Cancer Society
AICR: American Institute for Cancer Research
COM-B: Capability, Opportunity, Motivation-Behavior
CVD: cardiovascular disease
LE8: Life’s Essential 8
MEPA: Modified Mediterranean Eating Pattern for Americans
MVPA: moderate-to-vigorous physical activity
PA: physical activity
PRAPARE: Protocol for Responding to and Assessing Patients’ Assets, Risks, and Experiences
RAPA: Rapid Assessment of Physical Activity
RQA: rapid qualitative analysis
