# Supplementary material for: Assessment of Physical Activity Engagement and Promotion Among Cancer Survivors in Cardio-Oncology Care: Qualitative Study
Source: JMIR Cancer. 2026 Sep 11;12:e104332. doi: 10.2196/104332 (PMC13568692; doi:10.2196/104332)
Supplement: Supplementary file 1 [file cancer-v12-e104332-s001.pdf]

CVONC - Aim 1 Patient Interview Guide

Background

Thank you for agreeing to this interview. I am (NAME) from Ohio State University. We have invited you to participate because you have received cancer treatment that increases your risk for cardiovascular disease.

For this reason, we want to explore how an app could help you and your medical team track and manage your cardiovascular health to prevent disease. Tracking signs and symptoms help your medical team manage your care and can help inform patient-provider appointments.

There is currently no set way for your medical team to track and manage your cardiovascular health outside of the clinic, and often, signs and symptoms of cardiovascular health in cancer patients are missed. Because of this, we are developing a mobile application to help survivors such as yourself:

1. Track your signs and symptoms to manage your cardiovascular health
    a. **Symptoms are self-reported** and include things like fatigue, chest pain, or palpitations.
    b. On the other hand, **signs are often derived from devices** like blood pressure, weight, and activity levels.
2. Track and improve your physical activity to improve your cardiovascular health

During this interview, we want to hear your thoughts on these topics and how data from such an app and a physical activity intervention could assist you in managing your cardiovascular health. Of course, there are no right or wrong answers. It also means you are more than welcome to tell us if there is something that needs to be improved.

Voluntary Participation & Confidentiality

- The interview will take about 30-60 minutes. Before we begin the discussion, I want to ensure you understand that:
    - Your participation is completely voluntary. You can end the interview anytime. Your decision does not impact the care and treatment you receive.
    - The interview is confidential, meaning that your name will not be used in any reports or articles.
    - We will record the interview to collect data for our research. The recording will be transcribed and stored in a secured file on the OSUWMC network. It will not be used to identify you.
- Do you have any questions?

Demographic Questions

- First, may I ask your age, your gender, and your race and ethnicity?
- Could you please tell me a bit about yourself?

Clinical Details

1. Are you in active treatment, or have you already completed your treatment?
2. What type of cancer were you diagnosed with? When were you first diagnosed?
3. Have you or are you currently receiving treatment for your cancer?
4. When choosing a treatment option, did you learn of any potential risks to the heart associated with this treatment?

Engaging in Physical Activity

READ: Motivation refers to the internal factors that influence our decision-making and behaviors. This includes not just our goals but also habits, emotions, and decision-making.

- *Can you describe your reasons for engaging in physical activity?*
  - *Can you describe how this might change before, during, or after treatment?*
- *Can you describe why you may not want to engage in physical activity?*
- *Can you describe how this application could motivate you to engage in physical activity?*

READ: Opportunities refer to things outside of you that make engaging or maintaining activity possible.

These can be physical opportunities, like time, things, or places, or social opportunities, like family, friends, or your medical team.

- *Can you describe any opportunities that help you engage in activity or exercise?*
  - *In other words, are there any external factors (time, places, or tools) that help you engage in activity?*
- *Can you describe opportunities that make it hard to engage in activity or exercise?*
  - *In other words, are there any external factors (time, place, or tools) that make it hard for you to engage in activity or exercise?*
- *Can you describe how an app could help you be active?*

READ: The next questions are about your ability to engage in physical activity or exercise.

When we think of ability, we can think of physical abilities, like strength or stamina, or mental abilities, like knowing how to or remembering to do something.

- *Can you tell me about your current activity patterns?*
- *Can you describe any mental or physical abilities that help you engage in activity or exercise?*
- *Can you share tips for success for starting an exercise program or becoming more active?*
- *What challenges or barriers make regularly engaging in activity or starting a new exercise program hard?*
  - *How have you overcome them?*
- *How could an application help you become more active or exercise?*

Experience with prior apps

- **Can you tell me about any apps or online resources that help you exercise or increase your activity?**
  - **Probe:** Can you tell me about any app(s) or features included in that app that you **enjoyed**?
  - **Probe:** Can you tell me about any app(s) or features included in that app that you **disliked**?

Features *(if time permits)*

CVONC - Aim 1 Patient Interview Guide

Below is a list of app features identified through previous research. You are the expert, so we want your thoughts on the most important features we could include in an app to help you track your symptoms and increase your activity levels. Please rank the following features from most to least important and explain why (in-person, use note cards; if virtual, use Miro).

1. Ability to track symptoms occurrence.
2. Tracking specific symptom attributes (frequency, intensity, type, time)
3. Seeing trends in symptoms: Weekly, monthly, yearly.
4. Use the app or downloadable report to communicate symptoms with the medical team.
5. Access a community of other patients/survivors in similar situations (i.e., cancer patients at increased risk for cardiotoxicity).
6. Educational resources (e.g., education on symptom management, risk reduction strategies like Life's Essential 8, etc.)
7. A physical activity program to improve cardiovascular health

Cardiotoxic Symptoms *(if time permits)*

We are including the capability to track the following cardiotoxic symptoms in our app. Please rank the following **symptoms** from the most important to the least from your perspective:

1. Chest pain/tightness
2. Shortness of breath
3. Heart palpitations
4. Abnormal heart rate
5. Abnormal (high/low) blood pressure
6. Edema
7. Lightheadedness
8. Syncope
9. Excessive fatigue
10. Other: ______

Educational Resources *(if time permits)*

[Interviewer Script] We are considering including educational resources in our app. What information would be most helpful if the app included educational content?

Please rank the following **educational resources** from the most important to the least:

1. Exercise program to improve cardiovascular health and reduce symptom burden.
2. Descriptions of symptoms, treatments, and risks.
3. Tips to improve cardiovascular health (e.g., life's essential 8).
4. Understanding of how cancer works and how the cardiovascular system or heart functions.
5. Other: ___________________

Wrap Up

Thank you for your participation! You will be receiving a digital gift card in your inbox.

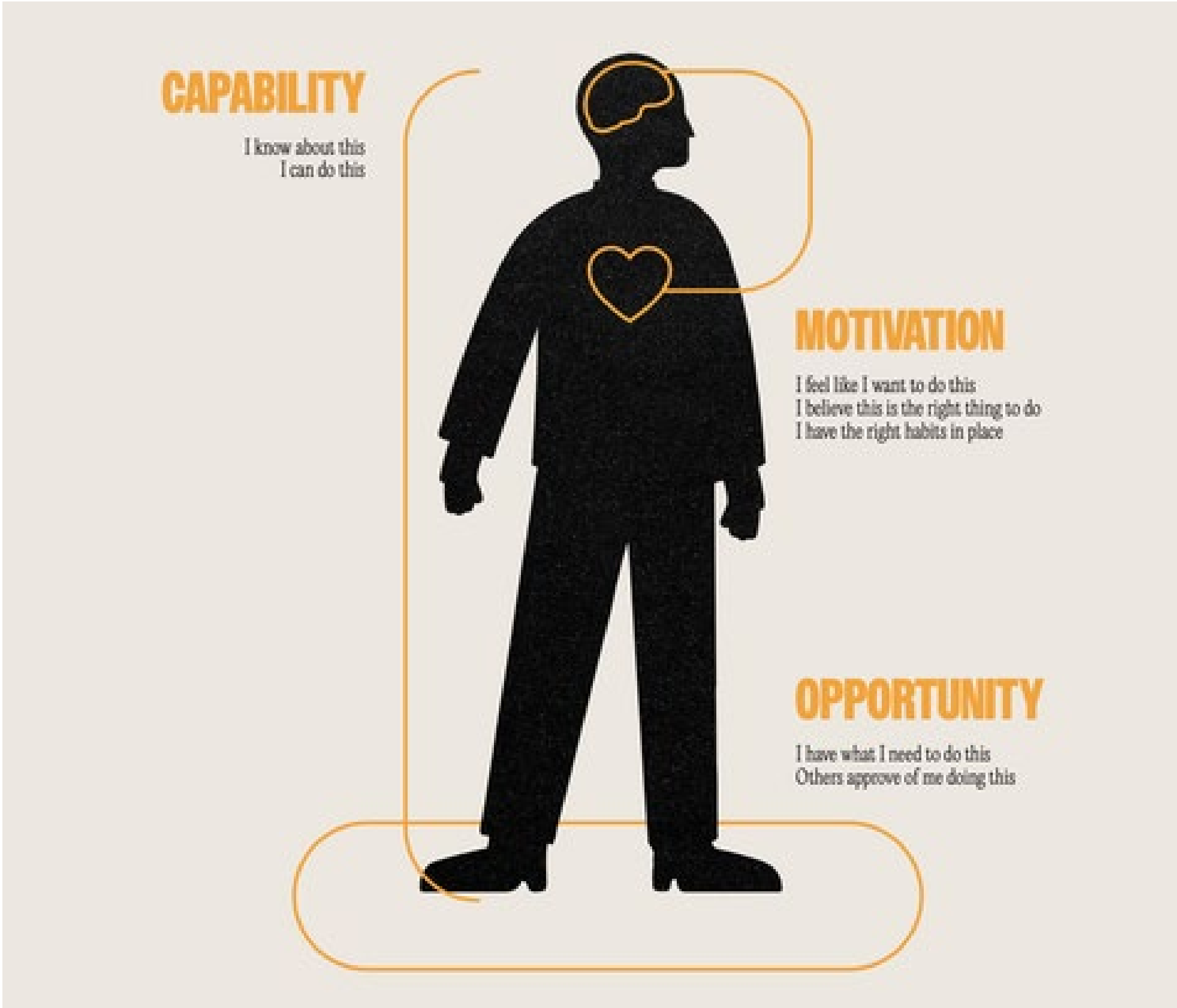

https://togetheragency.co.uk/news/the-com-b-model-of-behaviour-change-explained

Aim 1 - SME Interview Guide

Background

Thank you for agreeing to participate in this interview. I am (NAME), and I am from The Ohio State University.

We are developing a mobile application for cancer patients to 1) track of cardiotoxic symptoms and cardiovascular risk factors, and 2) promote physical activity. During this interview, we want to hear your thoughts on these topics, and how data from such an app and a physical activity intervention could assist you in caring for these patients.

The patients we are talking about include adults with a cancer diagnosis who are or have undergone a cancer regimen that includes a target, biologic, radiation, and immune-based therapy (e.g., TKIs and /or ICIs).

The **American Heart Association** has put together **Life's Essential 8**, the **key health behaviors and factors related to cardiovascular health.**

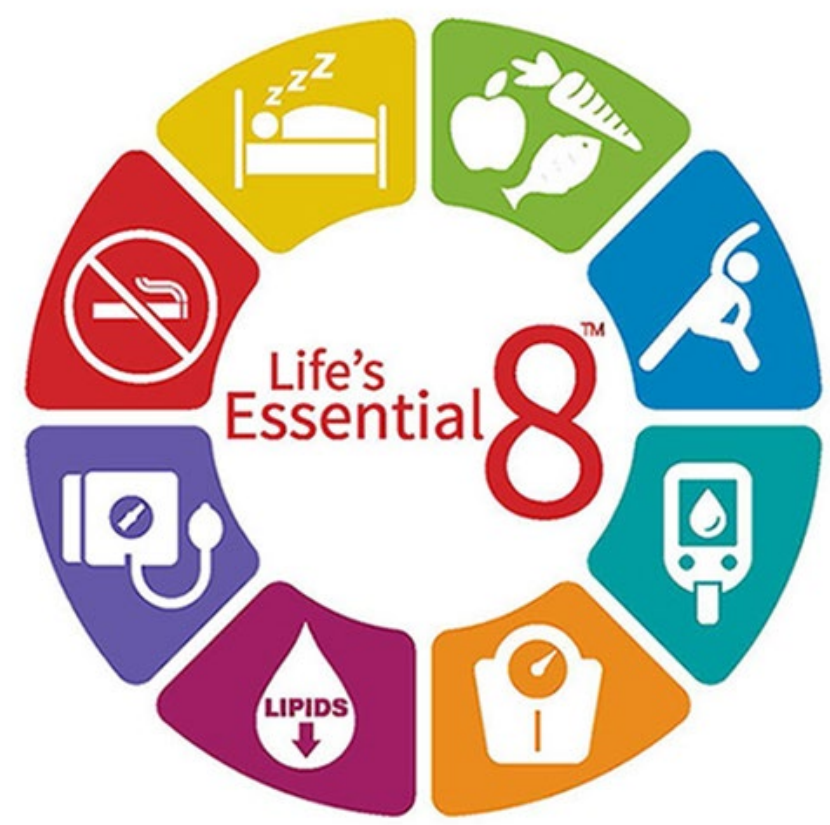

- Better cardiovascular health helps lower the risk of heart disease, stroke, and other major health problems in which you may be at risk due to cancer-related treatments.
- Regularly engaging in physical activity, one of the eight recommendations, can positively affect the many of these cardiovascular risk reduction factors (smoking, weight, cholesterol, blood sugar, blood pressure, sleep).

For this reason, we want to explore the idea of coupling this app with a **physical activity program** to help cancer patients' reduce their risk for cardiovascular disease.

Voluntary Participation & Confidentiality

- The interview will take about 60 minutes. Before we begin the discussion, I want to ensure you understand that:
  - Your participation is completely voluntary. If you choose to talk with me, you may end the interview anytime. Your decision does not impact in any way your job or employment status.
  - The interview is confidential. Your name will not be used in any reports or articles.
  - We will record the interview for data collection for our research. The recording will be transcribed and stored in a secured file on the OSUWMC network. The recordings will not be used to identify you in any way.
- Do you have any questions about this interview process?

Interview Questions

1. Can you state your name, current role, and how long you have been in this role?
2. Can you also tell us your age, your gender, and your race and ethnicity?

Job details

1. What type of cancer patients do you work with?
2. Tell us more about the role that cardiotoxicity plays when considering treatment options for cancer patients.

Symptom Tracking, Managing Cardiovascular Health, and Promoting Physical Activity		
**COM-B Model Components**	**Theoretical Domain Framework (TDF) Domains**	**Interview Questions & Prompts**
Psychological Capability	Knowledge	• Can you tell me about cardiotoxic symptoms cancer patients experience?<br>○ **Prompt:** When do they occur, how often, their intensity?<br>• What role does physical activity play in the lives of these cancer patients?<br>• Can you tell me how cancer patients manage their cardiovascular health?<br>○ **Prompt:** Do they follow the guidance of the AHA Life's Essential 8?
	Memory, attention, and decision processes	• When was the last time you wanted to track a cancer patient's symptoms related to cardiotoxicity? How did you go about this?<br>• When was the last time you recommended physical activity to a patient?
	Behavior Regulation	• What can you tell me about the relationship between cardiotoxic symptoms and physical activity?<br>○ **Prompt:** how does one affect the other?<br>• What role does physical activity play in a cancer patient's life?<br>○ **Prompt:** What are your thoughts on a **digital physical activity program** to help cancer patients manage their cardiovascular health?
Physical Capability	Physical Skills	• Can you describe in more detail how you keep track of your patient symptoms?<br>○ **Prompt:** How could this be improved or made useful?<br>• Can you describe in more detail how you help your patients' manage your cancer patients' cardiovascular health outside the clinic?<br>○ **Prompt:** How can you be confident that you understand your patient's cardiovascular health or life's essential 8 factors?
Social Opportunity	Social Influences	• Is there anyone to help track patient symptoms?<br>○ **Prompt:** Who could be of help and how?<br>• Is there anyone to help cancer patients' engage in physical activity?<br>• To what extent do the existing resources, if any, **help or hinder** you in tracking patient symptoms or promoting physical activity?
Physical Opportunity	Environmental context and resources	• Beyond people, is there anything in the environment that could **help or hinder** you in tracking patient symptoms? What about in promoting physical activity outside the clinic?<br>○ **Prompt:** Here are some examples, lack of time, using a journal, or an application
Reflective Motivation	Optimism	• (Ask if Time Permits) Are you confident that any barriers you may have to tracking patient symptoms can be solved?
	Intentions	• (Ask if Time Permits) Do you intend to keep track of your patients' symptoms?<br>○ **Prompt:** How so?<br>• (Ask if Time Permits) Do you intend to keep track of your patients' cardiovascular health risk factors (Life's Essential 8)?

	Beliefs about Consequences	• What do you think will happen if patients use an app to track patient symptoms? What about to track health behaviors and factors in Life's Essential 8?<br>• What are your thoughts of a physical activity intervention to improve CV health in cancer survivors?<br>○ **Prompt:** Discuss any benefits or consequences to track patient symptoms using an app?
	Goals	• What is your goal for your patients in terms of:<br>- *Symptom tracking*<br>- *Physical activity*<br>- *Cardiovascular health management*

Let's talk about the apps you use or have used and your experience.

Developing the app

1. What features would be most helpful if patients were to use an app to help them understand and manage their cardiovascular health?
    a. Prompts: Tracking vitals, symptoms, education, others?

[Note: If the participant does not like apps or is not savvy] I understand you may be hesitant to use apps to track patient health, but let's focus on the information and features you would find helpful in an app.

Our previous work explored developing an app to track cardiotoxic symptoms in cancer patients. Below is a list of features identified through previous research, but you are the expert, so we would like to get your thoughts on which, in your opinion, are the most important. Please rank the following and explain why (in-person use note cards; if the interview is held virtually, we will use Miro).

Communicating symptoms with Medical Team

1. What information about symptom burden do you rely on your patient to tell you?
2. What would it look like if the app could create a report of the patient's cardiotoxic symptom burden and cardiovascular health (Life's Essential 8) for the medical team?
    a. **Prompt:** What pieces of information would you like to see?
    b. **Prompt:** How could this help with clinical care? How could it inform patient visits?
    c. **Prompt:** What would make it useful?
    d. **Prompt:** How would you like to receive this report? (e.g., paper, email, EPIC in-basket)

Wrap Up

- Thank you for your participation!
