# Supplementary material for: Assessment of Physical Activity Engagement and Promotion Among Cancer Survivors in Cardio-Oncology Care: Qualitative Study
Source: JMIR Cancer. 2026 Sep 11;12:e104332. doi: 10.2196/104332 (PMC13568692; doi:10.2196/104332)
Supplement: Supplementary file 2 [file cancer-v12-e104332-s002.docx]

### **Sample Summary Matrix**

| **Type of Participant** | **# of Participants Endorsing** | **Participant IDs** | **COM-B Domain; Subdomain** | **Theme** | **Facilitator/Barrier** | **Representative Quote(s)** |
| --- | --- | --- | --- | --- | --- | --- |
| Survivor | 11 | 1-6, 8-12 | Capability; Physical | Current engagement in activity | Facilitator | S2: "I try to get up, try to get out, and try to get moving..." |
| Medical Team Member | 9 | 1,2,4,5,7-9,11,12 | Opportunity; Social Opportunity | Supportive social environments for survivors | Facilitator | MT8: “…their families are the biggest cheerleader…” |
| Survivor | 8 | 1-5, 7, 10, 11 | Motivation; Reflective Motivation | Physical Limitations | Barrier | S5: “I was planning on exercising this morning, but I've been a little bit under the weather…” |

### **Sample Participant Matrix**

| **Participant ID** | **COM-B Domain** | **Subdomain** | **Theme** | **Facilitator/Barrier** | **Representative Quote(s)** |
| --- | --- | --- | --- | --- | --- |
| Survivor 2 (S2) | Capability | Physical | - Active before cancer - Has previous activity knowledge - Is currently active | Facilitator | S2: "I try to get up, try to get out, and try to get moving..." |
| Medical Team Member 2 (MT2) | Capability | Physical | - Survivor self-efficacy | Facilitator | MT2: “You want to build a habit for them to do it on their own and do it with confidence.” |
