# Supplementary material for: Assessment of Physical Activity Engagement and Promotion Among Cancer Survivors in Cardio-Oncology Care: Qualitative Study
Source: JMIR Cancer. 2026 Sep 11;12:e104332. doi: 10.2196/104332 (PMC13568692; doi:10.2196/104332)
Supplement: Supplementary file 3 [file cancer-v12-e104332-s003.docx]

**Multimedia Appendix 3**

**Table S1.** Physical Activity and Cardiovascular Health Characteristics of Survivor Participants (n = 12)

| **Measure** | **Survivors (n = 12)** |
| --- | --- |
| Self-Report Meeting PA Guidelines (≥150 min. MVPA), n (%) | 5 (41.7%) |
| **Aerobic Physical Activity (RAPA)** |  |
| Physical activity score, mean (SD) | 5.2 (1.7) |
| Active/Meeting PA Guidelines n (%) | 6 (50%) |
| Underactive, n (%) | 6 (50%) |
| **Strength/Flexibility (RAPA)** |  |
| Both, n (%) | 5 (41.7%) |
| One only, n (%) | 4 (33.3%) |
| Neither, n (%) | 3 (25%) |
| **Modified LE8-Derived Health Score** |  |
| Standardized composite score (6 domains), mean (SD) | 71.3 (11.9) |
| Composite score, median (IQR) | 69.2 (64.8–77.9) |
| **LE8 Domain Scores, mean (n with data)** |  |
| Diet | 52.1 (12) |
| Physical Activity | 71.7 (12) |
| Nicotine Exposure | 91.7 (12) |
| Sleep | 89.2 (12) |
| Body Mass Index | 64.2 (12) |
| Blood Pressure | 59.2 (12) |
| Blood Lipids (supplementary, not in composite) ^a^ | 85.0 (4) |
| Blood Glucose ^b^ | N/A (unavailable for all participants) |
| **Self-Rated General Health, n (%)** |  |
| Excellent / Very Good | 3 (25%) |
| Good | 5 (41.7%) |
| Fair / Poor | 4 (33.3%) |

*Abbreviations: LE8, Life's Essential 8; RAPA, Rapid Assessment of Physical Activity; SD, standard deviation; IQR, interquartile range; PA, physical activity; MVPA, Moderate to Vigorous Physical Activity.*

*Note: Data reflect survivor participants only (n=12); medical team participants were not asked these items. Income, education, and employment were collected as part of the Protocol for Responding to and Assessing Patients' Assets, Risks, and Experiences (PRAPARE) survey.*

*Note: Self-Report Meeting PA Guidelines reflects a continuous self-reported item (weekly minutes of moderate-or-greater intensity activity, collected as part of the LE8 physical activity domain) classified against the ACS/AICR guideline of ≥150 minutes/week.*

*RAPA reflects a categorical classification based on the RAPA instrument, in which a score of 6 or higher (endorsement of item 6 or 7) indicates 'active' status.* *Strength and flexibility items are scored separately within RAPA and do not contribute to the aerobic score (1-7) or the study's classification of active status.*

*ᵃ* *Blood lipid data were available for 4 of 12 participants (mean lipid domain score = 85.0) and are reported descriptively; this domain was not included in the primary composite. A sensitivity analysis comparing composite scores with and without the lipid domain among these 4 participants showed a mean change of 1.0 points (range: −3.1 to +4.4).*

*ᵇ Blood glucose data were unavailable for all participants and were excluded from composite scoring.*

**Table S2.** Detailed Sociodemographic Category Breakdowns

| **Variable** | **Survivors (n = 12), n (%)** |
| --- | --- |
| **Annual Income** |  |
| $50,000-$74,999 | 4 (33.3%) |
| $75,000-$99,999 | 1 (8.3%) |
| $100,000-$199,999 | 5 (41.7%) |
| Prefer not to answer | 2 (16.7%) |
| **Education Completed** |  |
| High school diploma or GED | 1 (8.3%) |
| More than high school diploma/some college | 1 (8.3%) |
| College degree | 3 (25%) |
| Graduate or professional degree | 7 (58.3%) |
| **Employment** |  |
| Full-time | 1 (8.3%) |
| Part-time | 0 (0%) |
| Unemployed | 1 (8.3%) |
| Retired or disabled | 10 (83.3%) |

*Note: Data reflect survivor participants only (n=12); medical team participants were not asked these items. Income, education, and employment were collected as part of the Protocol for Responding to and Assessing Patients' Assets, Risks, and Experiences (PRAPARE) survey.*
