# Supplementary material for: Assessment of Physical Activity Engagement and Promotion Among Cancer Survivors in Cardio-Oncology Care: Qualitative Study
Source: JMIR Cancer. 2026 Sep 11;12:e104332. doi: 10.2196/104332 (PMC13568692; doi:10.2196/104332)
Supplement: Supplementary file 4 [file cancer-v12-e104332-s004.docx]

| **Domain** |  | **Theme** | **# of Participants**  ***S: Survivor***  ***MT: Medical Team*** | **Quote** | **Quote** | **Quote** |
| --- | --- | --- | --- | --- | --- | --- |
| **Physical Capability** | **Survivor Facilitators** | **Current Engagement in Activity** | 12  (S1-12) | *S2: "I try to get up, try to get out and try to get moving instead of just sitting in the chair all day and nothing, you know, watching life go by."* | *S3: "I mainly exercise my legs at least 4 days a week, …I do my upper body, and then 2 days a week, I dedicate to ab work, which you still can't see my abs, but they're there."* | *S7: "I'm more of a person that takes on the chores of life. And that's where I get a lot of my physical activity in."* |
|  | **Provider Facilitators** | **Survivor Self-Efficacy** | 3  (MT2, 8, 11) | *MT2: “You want to build a habit for them to do it on their own and do it with confidence.”* | *MT8: “And also it's a lot easier, I feel, to progress patients who maybe used to be athletes… compared to my patients who have always been sedentary, makes it pretty hard to get started when they're already at a pretty low point in their health.”* |  |
|  | **Survivor Barriers** | **Physical Limitations** | 12  (S 1-12) | *S2: "There's been incidences during the whole time where I couldn't be very active… I've seen a lot of muscle atrophy set in. I've got very weak and things like that."* | *S9: “I would just say the main, the main roadblock for me right now is just trying to not only weather my symptoms, but also not make them bigger than they are, and say, ‘Oh, I just took my 4 pills like I can't do anything for the next 4 hours’, which probably isn't true.”* | *S6: “It's physical limitations that mess you up…I just recently got shots in my knee and my one shoulder…like last week…I felt terrific. Saturday…I didn't even know how to get out of bed.”* |
|  | **Provider Barriers** | **Survivors’ Symptom- or Treatment-Related Burden** | 2  (MT 6, 7) | *MT6: “I think the exercise capacity goes down, and that limits the amount of exercise that they can do after the cancer treatment or during cancer treatment.”* | *MT7: “Every cancer patient is so different, and they have their own limitations and physical limitations.”* |  |
| **Psychological Capability** | **Survivor Facilitators** | **Knowledge and Awareness of Activity Levels and Ability** | 9  (S1-4, 6, 8, 9, 11, 12) | *S6: "I used to do Strong Women Stay Young or whatever, and that was just all, wait lists and stuff like that, but you did it 2 or 3 times a week.”* | *S4: “My husband could tell you I couldn't even walk to the very next condo…but every day I'd try to walk a little bit more, a little bit more, a little bit more, and gradually my strength came back….I could gradually do a little more, a little more and a little more. But you know that knowledge of the physical activity is what helped.”* | *S9: “Yesterday…I was able to mow most of the lawn and move around, and so I got in about 11,000 steps. So, I was happy about that…I know that, from a cardiovascular standpoint, I want to at least have a minimum baseline to…keep those good factors going for my heart health…so, I understand that as well.* |
|  | **Provider Facilitators** | **Knowledge and Awareness of Survivor Activity Levels and Ability** | 9 (MT 1, 2, 5-10, 12) | *MT8: “…if someone has no level of activity to start, I say, ‘I do not expect you to meet 150 minutes when we first start working together. We're going to slowly build it, because it's not attainable to do 30 min your first day and expect to have any energy the next day, and that eventually will be our goal.”* | *MT10: “I think we have a number of studies that show…it can improve overall survival being physically active…it can reduce the risk of the cancer coming back…I think it plays a huge role, and it's probably totally underutilized...”* | *MT8:“I can't predict how any of my patients are going to react to when they first start exercising. Some of my patients feel really great, and some of them, it makes them feel more crummy, and so we have to pivot and try other things first.”* |
|  | **Survivor Barriers** | **N/A** |  | | | |
|  | **Provider Barriers** | **Lack of Knowledge on Survivor Activity Levels** | 3  (MT 4, 5, 10) | *MT4: “I definitely ask them, ‘How many minutes are you walking, exactly?’, because patients can say, 'Yeah, I'm exercising.' But really, what does that mean compared to, you know, their baseline? Are they exercising more? How many, you know, days a week? Are they tolerating it?”* | *MT10: “If…they're walking…they started a mile, and then they went up to 3 or 4 miles a day. I mean, that would be super, super, helpful to know.”* | MT5: *“Having that metric of what is the actual goals they're achieving currently helps put together, kind of where you help direct them in the future.”* |
| **Physical Opportunity** | **Survivor Facilitators** | **Exercise Resources** | 11 (S 1-6, 8-12) | *S1: “I do think that like having things that you can do from the comfort of your own home is very important…if I'm super immunocompromised....But…for people that already belong to gyms, or like, have that community of people around them that, you know, are like, ‘let's go together,’ and they can kind of go together. All of those things would be very beneficial I think.”* | *S5: “We have an exercise bike and a treadmill in our basement…and it increases the opportunity because it's right there handy. And so that really helps me exercise. Because, yeah, I might drag myself out of my chair when I'm, when I'm, fatigued, but I know I only have to go downstairs.”* | *S3: “If the weather's adverse…I'll go to Y.M.C.A. and run. There's a track up there that I run on, so I have the opportunity to exercise every day somewhere.”* |
|  | **Provider Facilitators** | **Online Exercise Resources** | 4  (MT 1, 2, 4, 8) | *MT1: “A lot of my patients have been telling me that they have been using Youtube to find physical activity. There's been a couple of patients that say that they go on Youtube.”* | *MT2: “You could do classes on demand, you could do a whole, like, a channel…a whole library full of different classes that they can do, based on what they have at home. I mean, it gives them so much more access to do things at home… I can watch this video and see how to do an upper body session. And I don't even have to make it myself.”* | *MT4: “There's a lot of like videos online that people can watch but there's a lot of, you know, apps as well, that's kind of teach you, teach you different exercises. It's nice to have like a video that you can watch a short, very short, a simple video that kind of teaches you how to do exercises.”* |
|  | **Survivor Barriers** | **Weather and Time** | 7  (S 1, 3-7, 9) | *S7: "I’m very frustrated right now cause I would be doing a lot in the yard or planting more flowers… and now I can't do so."* | *S6: “I mean, it's harder in the wintertime to walk and stuff like that…That's the hard time. It's hard to walk.”* | *S5: “But for me, getting up and going, getting in a car and driving 15 to 20 minutes, to get dressed in your workout attire, and everything like that, and then come back home. That for me that is a negative.”* |
|  | **Provider Barriers** | **Lack of Access to Exercise Resources** | 9  (MT 1, 2, 4-8, 10, 11) | *MT2: “And accessibility to gyms and things that you know this side of town is very poor, so they maybe can't afford a gym, or they can't afford a treadmill home. So you have to find creative ways to keep them exercising on their own so that's gonna be the biggest barrier.”* | *MT5: “You can only do so much. I mean, I can't get them a pair of running shoes. I can't get them a membership at the gym, or make it warmer outside to run, or whatever it is that would actually work for them.”* | *MT8: “…if they want to go to a YMCA, or a gym, most of them say…it's a 25 or 30 minute drive at least, I live on dirt roads, we don't have sidewalks, I live on a farm that has very uneven ground that I don't feel safe walking on…a lot of environmental concerns...”* |
| **Social Opportunity** | **Survivor Facilitators** | **Supportive Social Environments (classes, family, friends)** | 12 (S 1-12) | *S9: “I have a couple of friends that I'll walk with from time to time, they don't live near me, so it's not a daily thing. But I mean, that's nice. And my wife's encouraging too.”* | *S4: “A lot of times my friends will call me and say, ‘Wanna walk to North Star Cafe,’…so it's a chance to talk and see people.”* | *S1: “And like Strava, like when you think about Strava, you like, track your friends, too, and you see what they're up to, or if anyone's near you, sometimes you can like, meet up with those people and do things together.”* |
|  | **Provider Facilitators** | **Supportive Social Environments for Survivors** | 9  (MT 1, 2, 4, 5, 7-9, 11, 12) | *MT2: “I do think like knowing that they're not alone and being in a community helps like it's a support system. So, it does kind of help them, you know, continue on. We, like, even we say like, if you have a buddy that you exercise with, you're more likely continue to do it.”* | *MT8: “…their families are the biggest cheerleaders, and they engage with them throughout the session. And then I have a lot of patients who are older and retired, who are part of exercise groups or walking groups and things like that. And so, their goal is to get back to being with those people.”* | *MT1: “…if they have somebody to encourage them or to work out with them, or, you know, do whatever, that's always helpful.”* |
|  | **Survivor Barriers** | **N/A** |  | | | |
|  | **Provider Barriers** | **N/A** |  | | | |
| **Reflective Motivation** | **Survivor Facilitators** | **Perceived Health Outcomes** | 12 (S 1-12) | *S8: “We were walking around all day, and for the last few years it's been me saying, ‘Oh, I'm tired. I can't walk that far,’…and then this time it was like my friends are trying to keep up with me...so I was able to hit my 10,000 steps like 4 days of that week, you know, and I haven't done that in forever.”* | *S1: “I feel like even in terms of physical appearance, again, there's so many things that change, but being able to still feel good about my body and like how I present myself is important to me.”* | *S5: “My motivation increased a few years ago, when I found I was a borderline diabetic, so that motivated me more.”* |
|  |  | **Exercise-Related Resources** | 7 (S 2-4, 6, 8, 9, 11) | *S3: “…[A Garmin] tracks my pace, distance, and…the primary reason I bought it was I wanted to keep track of my heart rate…I pay more attention to my heart rate than anything.”* | *S8: “I have a lot of apps on my iPad about exercise and different things, so those kind of help a little bit. I will open up one and say, ‘Okay, let me see if I can get a 10 minute workout in or a 5 minute workout in.’”* | *S11: “I always know I'm going to be going out and getting some physical activity. I have a big walking neighborhood, so I see my neighbors walking all the time, and it's like I guess I should go out and walk.”* |
|  |  | **Intentions to be Active** | 7  (S 2, 3, 5, 8, 9, 10, 11) | *S11: "Before that, I could bench press the 25 pounds. It's like, I wanna get back to that. I can do 20 now, but I can't do 25 yet."* | *S10: “A few years ago, I made a comment to my son that I was going to live to see my grandson graduate. My oldest grandson at the time…was ten. The problem is, after [redacted name], came [redacted name], who is only four. So…I have a goal there.”* | *S2: “And right now, the physical activity is to try and develop some core strength so I can walk without aids…so right now physical activity is to try and get that strength back so that I can move a little bit better.”* |
|  | **Provider Facilitators** | **Perceived Health Outcomes** | 7  (MT 1, 2, 4, 5, 8, 10, 12) | *MT4: “There have been studies that have shown they do better if they're very active throughout their treatment... The more active you are, you know, physically active, the better outcomes you can say that patients have. Whereas, if they start chemotherapy and are not as active then…they can develop clots… deconditioning and that could really affect their ability to get more treatment, and how they do.”* | *MT5: “It's extremely important. It's directly linked to their cardiovascular outcomes and their oncology outcomes. The more kind of fit they are from a cardiovascular perspective, the better they're gonna do in both arenas. So, the physical activity is critical.”* | *MT10: “A lot of times when patients are complaining about fatigue, I usually mention to them, you know, ‘Are you, are you being active at all?’ If not, I usually tell them to start slow and try to work your way up.”* |
|  |  | **Survivors’ Inclination to be Active** | 3  (MT 2, 3, 8) | *MT3: "So it's self-motivation. Most of the time I see that they are self-motivated. They want to be healthy, they want to pursue a healthy lifestyle, and they do that."* | *MT8: “Our thoracic oncology patients have very, very limited activity levels, and I've had patients look straight at me and say, 'That's all great info, and I understand. But I'm not going to do it.' And then I have other patients who it kind of, I think, lights a little bit of a fire under their butt and then they're all in. So, I've had both ends of the spectrum.”* | *MT2: "Anytime you exercise, people always seem to feel better afterwards. The hardest part is getting there. But once they start feeling better, they're more likely to continue so that's why they really enjoy and get better results for it.”* |
|  | **Survivor Barriers** | **Physical Limitations** | 8  (S 1-5, 7, 10, 11) | *S1: “I've just had severe back pain…like it's been miserable. So right now, it's mostly just walking with the dog around the block…I really liked swimming for a long time, because it was like less impact on my joints. And then biking for the same exact reason cause I have some cancer in my hip, and things are stiff.”* | *S2: “I'm fighting a lung infection, trying to get that taken care of, too. So that's kind of diminished the physical activity.”* | *S5: “I was planning on exercising this morning, but I've been a little bit under the weather, so I had to schedule a doctor's appointment. Well, that took out this morning. This afternoon…by the time I get those things done in the day, if I don't exercise one of the first things in the day, it won’t get done.”* |
|  |  | **Safety Concerns, Resource Preferences** | 3  (S 3, 4, 10) | *S4: "A lot of times during treatment, you're really afraid that you might hurt something or break something, that you're fragile and frail…You're worried about your heart rate going up too much or something like that, and you just feel weak."* | *S10: “But the only activities I don't engage in might involve the chance of me getting hurt in some way, or something like that.”* |  |
|  | **Provider Barriers** | **Physical Limitations/Treatment-Related Conditions** | 5  (MT 2, 5, 7, 8, 12) | *MT5: “I mean, there's limitations. They can feel pretty terrible going through chemo and things if they have any complications like myocarditis or pericarditis...”* | *MT7: “…this is a very sensitive time where I don't wanna push them through, you know, through fatigue, etcetera. And they are usually very much fatigued the first few months after transplant.”* | *MT8: “And I had another patient…and her goal was just to be able to wash her hair by herself and be able to put her hair up in a ponytail… So instead of doing like higher levels of activity, we were just focusing on very low-level shoulder mobility…so that she could still feel independent even at end of life.”* |
| **Automatic Motivation** | **Survivor Facilitators** | **Routine or Habit** | 7  (S 3-5, 8, 10-12) | *S4: “Just doing it as part of a routine. Say, this is my routine. I get up. I find the class that I want to go to at the center. I click on it and I go to it.”* | *S3: “I'm kind of a creature of habit…we set a time to run every morning, and we do it.”  “I have a set schedule in my mind…Every day I go in there. I don't refer to anything. I just know what I'm going to do, and I do it.”* | *S12: “But you've got to make that imprint on your mind that, hey, this is like brushing my teeth. I just, I just do it [exercise].”* |
|  |  | **Emotions Surrounding Physical Activity** | 5  (S 2, 4, 6, 7, 12) | *S4: “When I'm achy and stiff, I'll go to a stretching type class, and I'll just feel better. I also feel when I'm kind of groggy, I'll go to some of these classes and just getting my blood flowing…my mood improves. I just feel better.”* | *S2: “I also find that it’s kind of a feedback loop, being positive about it with a feeling of if I get exercise done…even if it's just like 10 minutes…it'll feed back to me where I'll feel better about things…it's that feedback loop.”* | *S12: “I just feel better…for about a year I was inactive…so being able to start doing, and having those [symptoms] go away and start doing stuff again really helps.”* |
|  | **Provider Facilitators** | **N/A** |  | | | |
|  | **Survivor Barriers** | **Emotions Surrounding Physical Activity** | 7  (S 1, 2, 6-9, 11) | *S1: “My body is so limited compared to what it used to be that I feel a lot of strong emotions about what I can't do anymore and I think that sometimes even subconsciously, just being like… I don't want to feel bad about myself right now, you know.”* | *S9: “Some people might call it laziness. just having a hard time inside getting started with something…It's not a specific thing I can put my finger on, but maybe just a lack of motivation to get into a real framework.”* | *S11: “When you’re tired, and you don’t want to go…it’s too easy to skip.”* |
|  | **Provider Barriers** | **Survivors’ Emotions Surrounding Physical Activity** | 3  (MT 1, 5, 8) | MT1: *“…the biggest thing is their own motivation, like if they're not motivated to exercise or to do any physical activity, they’re not gonna do it.”* | MT5: *“…Each person has to have a little bit of personal responsibility at some point to do it. But you can try to set them up for success…talking to them and educating them about the benefits of it.”* | MT6: *“You don't know what's happening in each person's world…you don't know what socioeconomic status they come from...whether they're able to pay the day-to-day bills…if the patient feels that he [provider] understands, they're more likely to follow through your recommendation.”* |
